# History of the Royal Academy of Sciences for the Year 1777; with the Mathematical and Philosophical Memoirs for That Year, Taken from the Registers of the Academy

**Published:** 1781-08

**Authors:** 


IV. *Histoire de l'Academie Royale des Sciences. Année* 1777. *Avec les memoires de mathematique et de physique, pour la meme année, tirés des Registres de cette Academie.* i. e. *History of the Royal Academy of Sciences for the year* 1777; *with the mathematical and philosophical memoirs for that year, taken from the Registers of the Academy.* 4to. Paris, 1780. 828 pages, with 14 copper-plates.

IN the historical part of this volume, which employs 154 pages, we find the eulogies of Jussieu, Bourdelin, and Haller, from each of which we mean to give extracts in some future number of our Journal. The memoirs that fall within the plan of our work, are as follows:

I. *Fifth memoir on Zinc, by* M. de Lassone.— The object of this paper is to examine the effects of caustic volatile alkali, fixed mineral alkali, lime water, and the acetous acid on this semi-metal. From a variety of experiments it appears, pears; that cauftic fixed alkalis (when concentrated) diffolve a part of the zinc, and reduce the remainder to a ftate of calx. Cauftic volatile alkali at firft did not feem to have any fenfible operation on the zinc, but after a time it attacked it in a manner very different from what fimple water would do, and the mixture was attended with a fingular phenomenon, particles of the zinc uniting with the glafs veffel in which it was digefted, and affuming a dark grey colour.

M. de Laffone obferves, that from every folution of zinc black flakes are feparated in greater or lefs abundance. Thefe flakes are the fame, whatever menftruum is employed. He finds that they are foluble in acids, and that alkalis precipitate them in the form of a white earth. He proves that they contain nothing of an inflammable or metallic nature, and he confiders them as an abforbent earth, which owes its black colour to a fmall portion of phlogifton it has retained.

The author concludes this paper with fome obfervations on the medicinal effects of zinc. Preparations of this femi-metal are employed in diforders of the eye; but inftead of the calx which is commonly ufed for this purpofe, he recomrecommends the acetous salt of zinc. He observes, that this salt, being more soluble in water than the acetous salt of lead, might be advantageously substituted in its stead. Flowers of zinc have been strongly recommended by Gaubius and others as a sedative remedy; but our author asserts, that although he has repeatedly prescribed them in his own practice, he never experienced any such effect from them; and he adds, that a physician of his acquaintance, of extensive practice, and who for several years gave the flowers of zinc in a variety of convulsive cases, and in larger doses than those recommended by Gaubius, never found them more powerfully sedative than the ordinary remedies to which this quality is attributed.

II. *A description of the nerves of the second and third cervical pairs. By* M. Vicq d'Azyr.

No part of anatomy has been more diligently cultivated by the moderns than neurology. To Walther, Senac, and Haller, we are indebted for minute accounts of the intercostal nerve; Meckel has immortalized himself by his description of the fifth and seventh pair of nerves; the nerves of the last cervical pair, which serve chiefly to form the great plexuses of the arm, and the nerves of the pelvis, have been carefully described by Camper, as those of the first cervical pair have by Asche; and M. Vicq d'Azyr in the paper before us gives us a very exact description of the second and third cervical pairs, the branches of which are very numerous. The accounts that have been hitherto given of these two pairs by anatomical writers are either erroneous or too concise. These were the author's reasons, as he informs us, for undertaking this task. Like Meckel, he has divided his dissertation into three parts; in the first he gives the remarks of the best authors on the subject; the second is employed in anatomical description; and in the third he explains the uses of these nerves.

III. *Of the combustion of Kunckel's phosphorus, and of the nature of the acid which results from this combustion.* *By* M. Lavoisier.

Since the discoveries made by M. Margraff with regard to phosphorus, this substance has been considered as a particular animal acid combined with phlogiston, in the same manner as sulphur is supposed to be formed by vitriolic acid united with that principle. From the experiments related in the present paper it appears, that this theory, though it may be well founded, is far from being complete.

M. Lavoiſier found that upon attempting to burn phoſphorus in a cloſe veſſel the quantity conſumed was ſmall compared with the maſs of air contained in the veſſel. This maſs was diminiſhed one fifth, and the acid produced by the combuſtion of the phoſphorus being collected, was found to exceed the weight of the phoſphorus employed in the experiment. This exceſs of weight was equal to the quantity of air abſorbed. The remaining air was unfit for reſpiration or combuſtion, but on adding to it a quantity of vital air produced by the reduction of precipitate *per ſe*, it reſumed all its properties of atmoſpheric air. Similar phenomena took place in the combuſtion of ſulphur. Hence M. Lavoiſier is inclined to conſider ſulphur as an acid deprived of this air, and the acid as a ſulphur to which it has been reſtored.

From theſe enquiries our author proceeds to conſider the combinations of the phoſphoric acid with alkaline and metallic ſubſtances. Upon being poured into lime water the mixture became turbid, and afforded a precipitate ſimilar to that produced by fixed air. The author obſerves, however, that the precipitates in theſe two caſes (notwithſtanding the reſemblance which ſome have ſuppoſed between the acid of phosphorus and fixed air) are essentially different; one being a chalk insoluble in water, and effervescing with acids; the other a soluble phosphoric salt liable to no such effervescence.

IV. *Of the means of improving the breed of Sheep. By* M. Daubenton.——The ingenious author of this paper has been employed for several years past, under the patronage of the intendant of the finances, in prosecuting a variety of interesting experiments for ascertaining the best means of improving the breed of sheep in France. For this purpose a numerous flock has been constantly under his direction since the year 1767; sheep have been procured from Rousillon, Flanders, England, Morocco, and even Thibet; and, in a word, no means have been neglected that could possibly promote the object in view.

M. Daubenton has from time to time communicated the result of his observations to the academy, and several of his papers on this subject have appeared in different volumes of their memoirs. In his present paper we meet with several curious remarks.

It is well known that, in general, by mixing different breeds of the same species we procure a mixture of the two, and a new breed, but the particular influence of the male or female female on these occasions seems hitherto to have escaped the attention of naturalists. M. Daubenton has observed, that in sheep, if we employ the female of the breed we mean to introduce, the wished-for improvement is not thoroughly obtained without a great number of crossings, whereas two are frequently sufficient to give the new breed its utmost degree of perfection, provided rams are employed instead of ewes; it being a fact that lambs of either sex have constantly a much greater resemblance to their father than their mother. Whether this observation holds good with other species of animals remains yet to be determined.

V. *Analysis of some waters brought from Italy by M. Cassini, jun. By* M. Lavoisier.—These waters were brought from the neighbourhod of Latera, famous for its mines of alum and sulphur. They proved to be of an aluminous nature, yet did not yield alum when evaporated, but only a saline mass with an excess of acid. M. Lavoisier found that by saturating this acid with an alkali, a precipitate was produced which was redissolved. When the saturation was complete the evaporation afforded chrystals of alum, and no marks of alkali remained. This confirms the observations made by Margraff and Macquer, that that the earth of alum is not a ſimple earth, but a combination of alkali with an argillaceous earth.

[ *To be continued.* ]

